# Determination of *IL-1B* (rs16944) and *IL-6* (rs1800796) genetic polymorphisms in IgA nephropathy in a northwest Chinese Han population

**DOI:** 10.18632/oncotarget.17603

**Published:** 2017-05-04

**Authors:** Daofa Zhang, Maowei Xie, Xiaohong Yang, Yin Zhang, Yan Su, Yanni Wang, Haiyang Huang, Hui Han, Wenning Li, Keying Fu, Huiluan Su, Wentan Xu, Yeguang Han, Ru Wang, Pei Zhang, Wei Wu, Yun Huang, Daojun Chen, Tianbo Jin, Jiali Wei

**Affiliations:** ^1^ Department of Nephrology, Hainan General Hospital, Haikou, Hainan 570311, China; ^2^ Central Laboratory, Hainan General Hospital, Haikou, Hainan 570311, China; ^3^ Key Laboratory of Resource Biology and Biotechnology in Western China, Northwest University, Ministry of Education, Xi'an, Shaanxi 710069, China; ^4^ Xi'an Tiangen Precision Medical Institute, Xi'an, Shaanxi 710075, China

**Keywords:** IgAN, *IL-1B*, *IL-6*, Chinese Han population, genetics polymorphisms

## Abstract

IgA nephropathy (IgAN) is the most common form of primary glomerulonephritis worldwide, but etiology and pathogenesis continue to be poorly understood. Polymorphisms in the cytokine genes may play a role in the etiology and pathogenesis of IgAN. The incidence of different between diverse ethnic groups suggested important genetic influences on its pathogenesis.

We genotype 10 single nucleotide polymorphisms (SNPs) in *IL-1B* and *IL-6* gene using Sequenom Mass-ARRAY technology from 417 IgAN patients and 463 healthy controls of the Chinese Han population. We evaluated these SNPs associated with IgAN utilising the chi-square tests and genetic model analysis.

We identified that the minor alleles of rs16944 (“A”), rs1800796 (“G”) in *IL-1B, IL-6* were involved in an increasingly risk of IgAN in allelic model analysis, respectively. The rs16944 in *IL-1B* and rs1800796 in *IL-6* were associated with 1.23-fold (95% CI, 1.02-1.48, P = 0.031) and 1.33-fold (95% CI, 1.11-1.66, P = 0.003) increases in the risk of developing IgAN, respectively. There was only rs1800796 still correlated with IgAN in the allelic model after adjustment by age and gender and the Bonferroni correction. In addition, Haplotype G_rs1800796_A _rs2069837_G _rs2069840_ (*P* = 0.037) and G _rs1800796_A _rs2069837_C _rs2069840_ (*P* = 0.042) in *IL-6*were considered to be associated with increased IgAN risk.

This study verified the *IL-6, IL-1B* genetic variants polymorphisms contributed to IgAN susceptibility in a Chinese Han population. Although we identified SNPs susceptibility, however, replication studies and functional research are required to confirm the genetic contribution in IgAN.

## INTRODUCTION

Berger and Hinglais firstly described IgA nephropathy (IgAN) in 1968, and it is a relatively newly recognized disease [1, 2]. In the next few decades, IgAN was considered as a common glomerulonephritis. At the initial time of the diagnosis of long-term prognosis, predicting is still difficult in this disease. There were four processes are as follows induce the renal injury in IgAN: (1) aberrant glycosylation of IgA1; (2) synthesis of antibodies directed against galactose-deficient IgA1; (3) binding of the galactose-deficient IgA1 by the anti-glycan/glycopeptide antibodies to form immune complexes; (4) accumulation of these complexes in the glomerular mesangium to initiate renal injury. Others consensual factors [3–5] include the occurrence of arterial hypertension, presence of severe lesions on initial renal biopsy, such as focal and segmental hyalinosis, and tubulointerstitial fibrosis. There also exist some other factors, such as age [6], sex, obesity or overweight, hyperuricemia or hypertriglyceridemia [7], and different immune genetic markers, all of the which are not extensively confirmed or controversial.

In IgAN patients, a previous study reported that Interleukin-6 (*IL-6*) contributed to the proliferation of cells [8]. *IL-6* encodes a cytokine that functions in inflammation and the maturation of B cells. And the encoded protein has turned out to be an endogenous pyrogen can induce fever in people who carried the infections or autoimmune diseases. The functioning of *IL-6* is associated with a variety of disease states, such as diabetes mellitus and systemic juvenile rheumatoid arthritis.

A study involved to patients with a progressive clinical course has reported that Increased *IL-6* glomerular expression as well as urinary excretion [9]. It has been suggested to be a prognostic marker for IgAN, but its role is still debated [10, 11]. At tumor sites, inflammatory cytokines can modulate the growth and invasive properties of tumor cells, and *IL-1* is part of the most potent pro-inflammatory cytokines [12]. *IL-1* exists in two agonistic forms, *IL-1*alpha and *IL*–beta (*IL-1B*). *IL-1B* coding for protein *IL1-β* is located in the 2q14 chromosome, has 7,153 kb and seven encoding regions. *IL-1B* plays a key role in the regulation of the immune responses and in the production of a variety of inflammatory mediators, together with other molecules [13].

In this study, we conducted an extensive association analysis to assess the roles of *IL-1B* and *IL-6* polymorphisms and haplotypes on susceptibility to IgAN in a Chinese Han population from a case-control study. A total of 10 single nucleotide polymorphisms (SNPs) were studied in this association analysis in an attempt to identify markers that might guide intervention decisions and improve patient survival.

## RESULTS

A total of 880 participants, including 417 IgAN patients and 463 controls, were analyzed in this study. Basic patient characteristics (gender and age) are listed in Table 1. 31.0% of the IgAN patients were men and 16.4% were women, while 30.1% of the controls were men and 22.5% were women. The mean ages were 33.22 ±12.15 years (mean ± SD) for IgAN patients and 50.65 ±11.79 years for controls.

**Table 1:** Characteristics of cases and controls in this study

Variable	Cases	Controls	P-Value
	417(47.4%)	463(52.6%)	
Gender			< 0.012 (2-tailed)
Male	273(31.0%)	265(30.1%)	
Female	144(16.4%)	198(22.5%)	
Age, yr (mean ±SD)	33.22±12.15	50.65 ±11.79	< 0.000 (2-tailed)

10 SNPs in the *IL-1B* and *IL-6* gene were genotyped in the IgAN patients and the controls. Table 2 listed the basic characteristics of these SNPs in the study population. All of the ten SNPs have been conformed to HWE in the control group (*P* > 0.05), and two-sided Pearson chi-square tests were used to identify differences in allele frequency distributions between IgAN patients and controls.

**Table 2:** Frequency distributions of *TERT* alleles and their associations with IgAN risk

SNP ID	Position	Gene	Role	Allele A*/B	MAF		HWE *P*	OR (95% CI)	*^a^P*	*^b^P*
					Cases	Controls				
rs2853550	113587121	*IL-1B*	Downstream	A/G	0.115	0.087	0.067	1.37(1.00-1.87)	**0.050**	0.50
rs1143643	113588302	*IL-1B*	Intron	C/T	0.498	0.474	0.191	1.1(0.91-1.33)	0.320	1
rs3136558	113591275	*IL-1B*	Intron	G/A	0.371	0.368	0.617	1.01(0.83-1.23)	0.890	1
rs1143630	113591655	*IL-1B*	Intron	T/G	0.183	0.159	1	1.19(0.93-1.53)	0.169	1
rs1143627	113594387	*IL-1B*	Promoter	G/A	0.528	0.481	0.160	1.2(1.00-1.45)	0.053	0.530
rs16944	113594867	*IL-1B*	Promoter	A/G	0.528	0.476	0.162	1.23(1.02-1.48)	**0.031**	0.310
rs1143623	113595829	*IL-1B*	Promoter	G/C	0.435	0.401	0.286	1.15(0.95-1.39)	0.149	1
rs1800796	22766246	*IL-6*	Promoter	G/C	0.354	0.288	0.309	1.35(1.11-1.66)	**0.003**	**0.030**
rs2069837	22768027	*IL-6*	Intron	G/A	0.216	0.189	0.097	1.18(0.94-1.49)	0.161	1
rs2069840	22768572	*IL-6*	Intron	G/C	0.109	0.083	0.116	1.35(0.98-1.85)	0.066	1

**Abbreviations:** SNP = single nucleotide polymorphism, CI = confidence interval, HWE = Hardy-Weinberg Equilibrium, MAF = minor allele frequency, ORs = odds ratios.

**Notes:** *Minor allele; ^a^*P* were calculated from two-sided Pearson chi-square tests for either allele frequency and ^b^*P* were adjusted by Bonferroni correction; *p*-value < 0.05 indicates statistical significance.

The rs2853550 and rs16944 in *IL-1B*, and rs1800796 in *IL-6* were associated with 1.37-fold (95% CI, 1.00-1.87, *P* = 0.050), 1.23-fold (95% CI, 1.02-1.48, *P* = 0.031), and 1.33-fold (95% CI, 1.11-1.66, *P* = 0.003) increases in the risk of developing IgAN, respectively. The SNP rs2853550 had a weak significant difference. Only one (rs1800796) of the three SNPs was still correlated with IgAN in the allelic model after Bonferroni correction.

And then, we determined the association between these 10 SNPs and IgAN risk using four genetic models (Table 3); *P*-values were calculated using the chi-square test. The “A/G-A/A” genotype at the rs2853550 SNP increased the risk of IgAN in the dominant model (OR = 1.46; 95% CI, 1.04-2.05, *P* = 0.030), and “A/G” in the overdominant model with a P = 0.023 (OR = 1.151; 95%CI, 1.06 – 2.15). The “G” allele in rs16944 decreased the IgAN risk in the log-additive model (OR = 0.80; 95% CI, 0.66-0.97, *P* = 0.026). However, these results were no statistical significance after adjustments for age and gender and the Bonferroni correction.

**Table 3:** Genotypic model analysis of relationship between SNPs and IgAN risk

SNP-ID	Model	Genotype	Case (n, %)	Control (n, %)	Without adjustment		With adjustment		
					OR (95% CI)	*P*	OR (95% CI)	*^a^ p*	*^b^ P*
rs2853550	Codominant	G/G	387 (84.1%)	327 (78.4%)	1	0.075	1	0.140	0.60
		A/G	66 (14.3%)	84 (20.1%)	1.51 (1.06-2.15)		1.55 (1.00-2.40)		
		A/A	7 (1.5%)	6 (1.4%)	1.01 (0.34-3.05)		0.80 (0.19-3.38)		
	Dominant	G/G	387 (84.1%)	327 (78.4%)	1	**0.030**	1	0.072	0.36
		A/G-A/A	73 (15.9%)	90 (21.6%)	1.46 (1.04-2.05)		1.47 (0.96-2.26)		
	Recessive	G/G-A/G	453 (98.5%)	411 (98.6%)	1	0.920	1	0.680	1
		A/A	7 (1.5%)	6 (1.4%)	0.94 (0.31-2.83)		0.74 (0.18-3.12)		
	Overdominant	G/G-A/A	394 (85.7%)	333 (79.9%)	1	**0.023**	1	**0.048**	0.24
		A/G	66 (14.3%)	84 (20.1%)	1.51 (1.06-2.15)		1.51 (1.06-2.15)		
	Log-additive	---	---	---	1.35 (0.99-1.83)	0.056	1.34 (0.92-1.97)	0.130	0.650
rs16944	Codominant	A/A	97 (20.9%)	110 (26.5%)	1	0.082	1	0.460	1
		G/A	247 (53.4%)	218 (52.5%)	0.78 (0.56-1.08)		0.83 (0.55-1.25)		
		G/G	119 (25.7%)	87 (21%)	0.64 (0.44-0.95)		0.74 (0.45-1.20)		
	Dominant	A/A	97 (20.9%)	110 (26.5%)	1	0.053	1	0.270	1
		G/A-G/G	366 (79%)	305 (73.5%)	0.73 (0.54-1.00)		0.80 (0.54-1.19)		
	Recessive	A/A-G/A	344 (74.3%)	328 (79%)	1	0.098	1	0.380	1
		G/G	119 (25.7%)	87 (21%)	0.77 (0.56-1.05)		0.84 (0.57-1.24)		
	Overdominant	G/G-A/A	197 (47.5%)	216 (46.6%)	1	0.81	1	0.84	1
		G/A	218 (52.5%)	247 (53.4%)	0.97 (0.74-1.26)		0.97 (0.69-1.34)		
	Log-additive	---	---	---	0.80 (0.66-0.97)	**0.026**	0.86 (0.67-1.10)	0.220	0.880
rs1800796	Codominant	C/C	239 (51.6%)	176 (42.4%)	1	**0.014**	1	**0.003**	**0.012**
		C/G	181 (39.1%)	184 (44.3%)	1.38 (1.04-1.83)		1.70 (1.19-2.43)		
		G/G	43 (9.3%)	55 (13.2%)	1.74 (1.11-2.71)		2.03 (1.17-3.53)		
	Dominant	C/C	239 (51.6%)	176 (42.4%)	1	**0.006**	1	**9.00E-04**	**3.2 E-03**
		C/G-G/G	224 (48.4%)	239 (57.6%)	1.45 (1.11-1.89)		1.77 (1.26-2.48)		
	Recessive	C/C-C/G	420 (90.7%)	360 (86.8%)	1	0.063	1	0.089	0.356
		G/G	43 (9.3%)	55 (13.2%)	1.49 (0.98-2.28)		1.57 (0.93-2.66)		
	Overdominant	C/C-G/G	231 (55.7%)	282 (60.9%)	1	0.12	1	**0.023**	0.115
		C/G	184 (44.3%)	181 (39.1%)	1.24 (1.10-1.62)		1.48 (1.05-2.07)		
	Log-additive	---	---	---	1.34 (1.10-1.63)	**0.004**	1.51 (1.18-1.94)	**0.001**	**0.004**

**Abbreviations:** CI = confidence interval, OR = odds ratio, SNP = single nucleotide polymorphism.

**Notes:** *p-*values were calculated by *Wald* test and *p-*value < 0.05 indicates statistical significance;

^a^*P* were adjusted by gender and age and ^b^*P* were adjusted by Bonferroni correction.

For the rs1800796 SNP, the “G” allele increased the risk 1.38-fold (95% CI, 1.04-1.83; *P* = 0.014) in the codominant model, the “C/G-G/G” genotype increased the risk of IgAN 1.45-fold (95% CI, 1.11-1.89; *P* = 0.006) in the dominant model, and the “G” allele increased the risk 1.34-fold (95% CI, 1.10-1.63; *P* = 0.004) in the log-additive model. In addition, after adjustment for age and gender, the “G” allele increased the IgAN risk 1.70-fold (95% CI, 1.19-2.43; *P* = 0.003) in the codominant model, the “C/G-G/G” genotype increased the risk of IgAN more than 1.70-fold (95% CI, 1.26-2.48; *P* = 9.00E-04) in the dominant model, and the “G” allele increased the risk more than 1.50-fold (95% CI, 1.18-1.94; *P* = 0.001) in the log-additive model. In the overdominant model, “C/G” increased the risk by 1.48-fold (P = 0.023) adjusted by gender and age. According to the adjusted by Bonferroni correction, mutations at rs1800796 (codominant *P* = 0.012; dominant P = 3.2 E-03; additive *P* < 0.004) were still associated with an increased IgAN risk.

**Figure 1:**
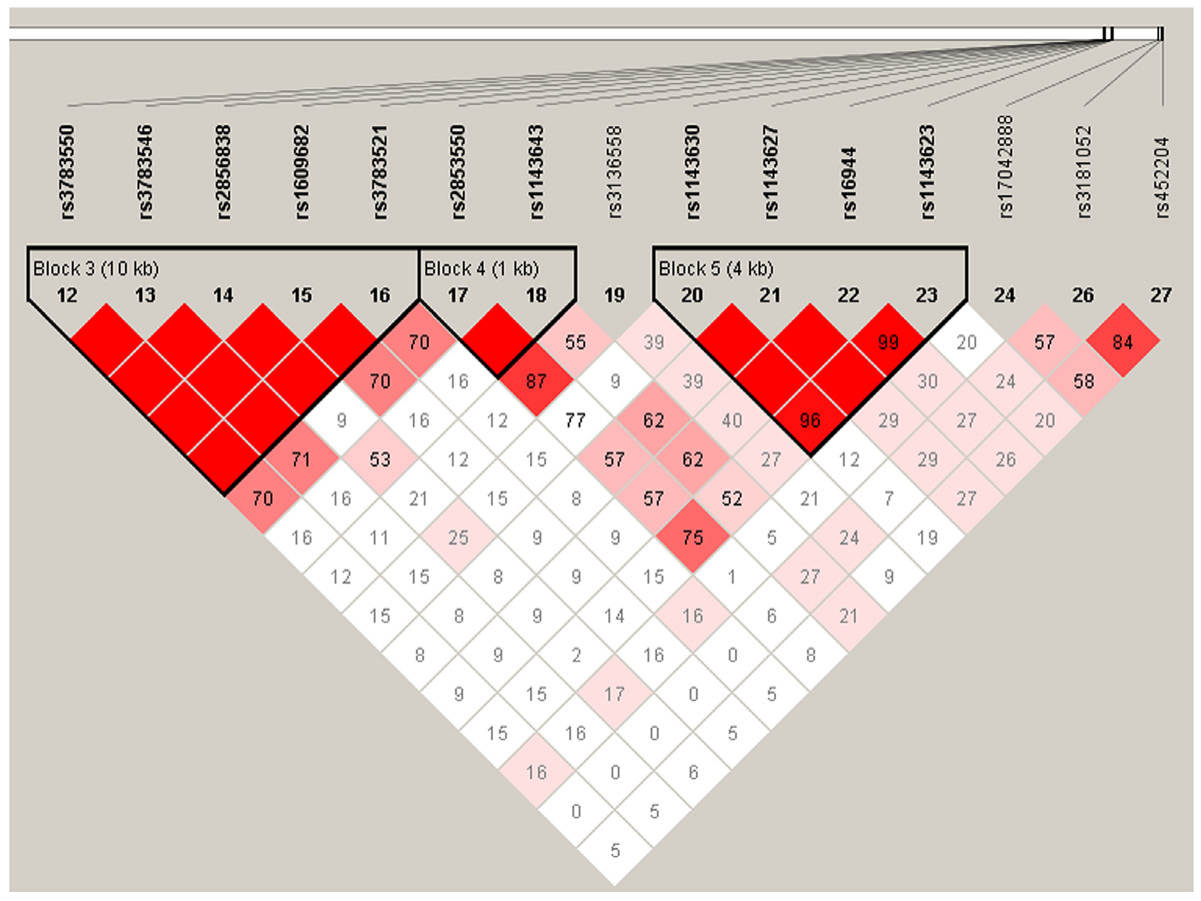
Linkage disequilibrium patterns of 7 SNPs in *IL-1B*

**Figure 2:**
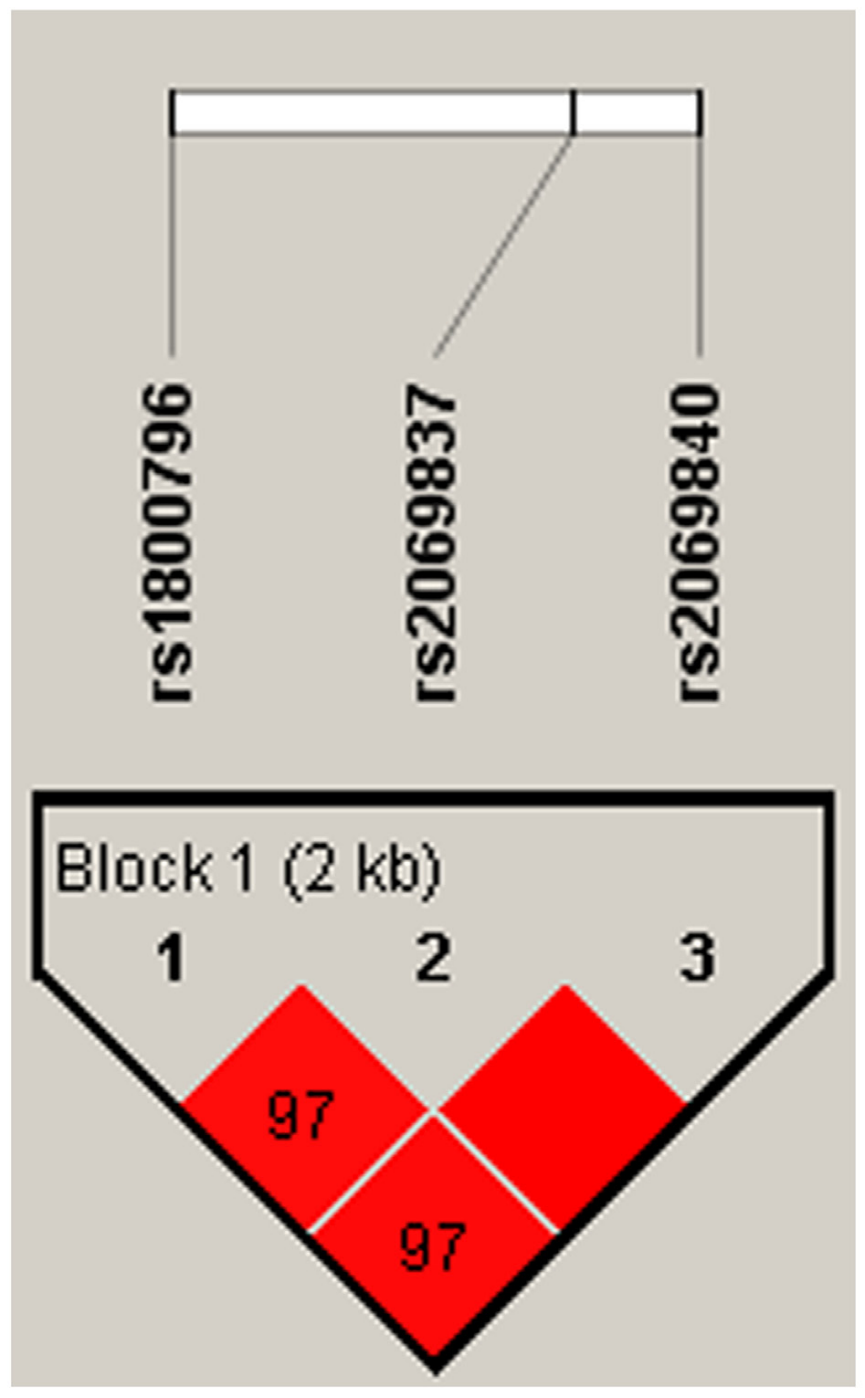
Linkage disequilibrium patterns of three SNPs in *IL-6*

*IL-1B* and *IL-6* genetic polymorphisms were characterized utilizing the haplotype and linkage disequilibrium (LD) analyses. LD was determined pairwise among all these 10 SNPs, the haplotype structure of the two genes were analyzed, two blocks (Figure 1) were detected in studied *IL-1B* SNPs and one block (Figure 2) was found in IL-6 SNPs by haplotype analyses.

Finally, a haplotype-based association study was performed to examine associations between *IL-1B* and *IL-6* haplotype and risk of IgAN (Table 4), and P-values were calculated using the Wald test. Altogether we detected that there were 11 haplotypes in the two genes and only two haplotypes (G_rs1800796_A _rs2069837_G _rs2069840_ and G _rs1800796_A _rs2069837_C_rs2069840_) were found to increase risk of suffering from IgAN by more than 1.40-fold (95 % CI, 1.02 - 1.95; *P* = 0.037) and 1.90-fold (95 % CI, 1.03 - 3.60; *P* = 0.042), respectively. After adjusting for age and gender, the “G_rs1800796_A _rs2069837_G _rs2069840_” (95% CI, 1.05 - 2.40; *P* = 0.027) and “G_rs1800796_A_rs2069837_C_rs2069840_” (95% CI, 1.09 - 5.02; *P* = 0.029) haplotypes increased the IgAN risk 1.59- fold and 2.34-fold, respectively. Interestingly, for “G_rs1800796_G_rs2069837_C_rs2069840_”, there existed a statistically significant difference (OR = 1.39; 95% CI, 1.04 - 1.87; *P* = 0.026) after adjusting for age and gender.

**Table 4:** Haplotype analysis results in this study

	rs2853550|rs1143643	Freq	Without adjustment		With adjustment	
			OR (95% CI)	*P*-value	OR (95% CI)	*P*-value
Haplotype	G| T	0.515	1	---	1	---
	G| C	0.385	1.04 (0.85 - 1.28)	0.680	1.02 (0.79 - 1.31)	0.900
	A| C	0.100	1.37 (0.99 - 1.88)	0.055	1.35 (0.90 - 2.01)	0.140
	rs1143630| rs1143627| rs16944| rs1143623					
	G| A| G| C	0.497	1	---	1	---
	G| G| A| G	0.247	1.15 (0.91 - 1.45)	0.250	1.08 (0.81 - 1.45)	0.590
	T| G| A| G	0.168	1.31 (1.00 - 1.71)	0.053	1.21 (0.86 - 1.69)	0.270
	G| G| A| C	0.082	1.39 (0.97 - 2.00)	0.076	1.33 (0.85 - 2.08)	0.220
	rs1800796| rs2069837| rs2069840					
	C| A| C	0.677	1	---	1	---
	G| G| C	0.198	1.25 (0.99 - 1.58)	0.062	1.39 (1.04 - 1.87)	**0.026**
	G| A| G	0.096	1.41 (1.02 - 1.95)	**0.037**	1.59 (1.05 - 2.40)	**0.027**
	G| A| C	0.025	1.92 (1.03 - 3.60)	**0.042**	2.34 (1.09 - 5.02)	**0.029**

**Abbreviations:** CI = confidence interval, OR = odds ratio, SNP = single nucleotide polymorphism.

**Notes:** *p*-values were calculated by *Wald* test and *p*-value < 0.05 indicates statistical significance;

*Adjusted by gender and age.

## DISCUSSION

The onset of IgAN may be associated with infections in the upper respiratory tract. Genetic analysis of familial IgAN is the most promising approach to the identification of IgA disease [14, 15]. For example, a recently study reported that 30 IgAN families were analyzed utilizing the whole-genome scanning [16]. However, in IgAN it is still not understood how the composition of circulating immune complexes affects the disease presentation, severity, and/or progression. Five novel variants were correlated with a protective effect against IgAN susceptibility were identified in a Genome-wide study [17]. It was reported that people carried protective alleles and had the lowest prevalence of IgAN in African populations. But in Asians, it was reported that people have fewer protective variants and have the highest prevalence. In a genome-wide linkage analysis study with 30 multiplex kindreds, had demonstrated the linkage of IgAN to 6q22–23 [16].

There is no doubt that there exist genetic components to the IgAN pathogenesis and IgAN clinical expression. In ourstudy, we systematically examined the impact of 10 SNPs in the *IL-1B* and *IL-6* loci on susceptibility to IgAN. We identified the association between SNPs and the susceptibility of esophageal cancer in five genetic models (codominant, dominant, recessive, overdominant and additive), which equivalent of repeated multiple comparisons, increasing the chance of making type I errors. Meanwhile, we should perform the *P* correction with Bonferroni correction. We found that the rs2853550, rs16944 in *IL-1B* and rs1800796 in *IL-6* genetic polymorphisms were correlated with an increased the IgAN risk in a northwestern Chinese Han patients population. To the best of our knowledge, this is the first case-control study to investigate this association. Persistent inflammation is linked with cancer development and progression [18]. *IL1-B*, a key cytokine released from glial cells, is critically involved in the pathogenesis of chronic pain [19], memory deficit [20], via activation of IL-1R1 [21]. It has revealed that treatment with *IL1B* can promote the invasiveness of breast cancer (BC) cells *in vitro* [22, 23]. A study reported that *IL1B* induced osteoprotegerin secretion, independent of BC subtype and basal osteoprotegerin levels [24]. The polymorphism rs1143630 is located in intron four, a noncoding region of the *IL1B* gene. Many articles refer to associations in intronic regions and risk of diseases [25]. The SNP rs1143630 of *IL1B* in the additive model considering the “T” allele as risk showed association with the preeclamptic event [26]. In our study, we detected that the SNP rs1143630 was not associated with IgAN risk. For rs1143633, there existed the significant difference between patients with schizophrenia and controls, and also found a association between rs16944 polymorphisms and female patients with schizophrenia in a Japanese population [27]. In this article, the results we get showed that rs16944 polymorphism increased in the risk of developing IgAN.

GQ Sun et al. [28] found that the *IL-6* rs1800795 G>C polymorphisms presented an increased risk in dominant and recessive models, but they did not find that the IL-6 rs1800796 CC were correlated with an increased risk of coronary artery disease. A study reported that the rs1800796 TT genotype was associated with increased the susceptibility of cerebral thrombosis in the Chinese population [29]. Previous meta-analysis demonstrated significant association between rs1800796 of IL6 and cancer risk, with the allele G as a risk allele [30]. The frequency of GG genotype of rs2069837 was higher in hepatocellular carcinoma patients, compared with controls (P < 0.05) [31]. In our current study, we for the first time studied the correlations between three SNPs (rs1800796, rs2069837 and rs2069840) in *IL-6* gene and IgAN susceptibility in a Chinese population. Rs1800796 SNP was associated with IgAN risk, rs2069837 and rs2069840 showed no correlations with IgAN susceptibility. Further studies should involve some others regions and ethnic groups. None of previous study found that IL-6 haplotype was directly associated with IgAN risk. Our study detected that haplotype “Grs_1800796_A_rs2069837_G_rs2069840_” and “G_rs1800796_Ars_2069837_C_rs2069840_” are related with the IgAN increasing risk.

Some limitations of the present study should be considered when interpreting the results. First, Several risk factors, such asgender, absence of macrohematuria, hypertension, severe proteinuria, smoking, and histopathologic changes, had been described to be associated with a poor outcome [32]. But in our articles, we only considered age and age, the further studies should collect more details of the clinical information. Second, the associations between the two genes (*IL-1B* and *IL-6*) polymorphisms and clinic pathological disease type were not evaluated in this study, Finally, both IgAN patients and controls were enrolled at the same hospital and therefore it may not be representative of the general population. Additional studies are needed to clarify the genetic mechanisms underlying IgAN by fine-mapping the susceptibility regions of the variants.

In conclusion, we demonstrated that the *IL-1B* and *IL-6* genetic polymorphisms are associated with an increasing risk of IgAN in a Chinese case-control population. This study offers new information on the relationship between two genes (*IL-1B* and *IL-6*) polymorphisms and IgAN susceptibility. Moreover, this study reveals the molecular markers associated with of IgAN susceptibility and could therefore be used as diagnostic and prognostic.

## MATERIALS AND METHODS

### Ethics and consent

All subjects were informed of the purpose of the study and the experimental procedures involved. The study protocol was approved by the ethics committee of the First Affiliated Hospital of Xi'an Jiaotong University. We also obtained signed informed consent from all participants. The experimental protocol was implemented in accordance with the approved guidelines.

### Study participants

From August 2012 to July 2016, 417 patients (273 males and 114 females, mean age of 33.22±12.15) were recruited to participates form Han population in the northwest China, all the cases were diagnosed as IgAN by renal biopsy and none-familialy IgAN cases. 463 healthy subjects (265 males and 198 females, mean age of 50.65 ±11.79) were recruited from routine healthy examinations in the same hospitals. All subjects were unrelated Chinese Han people living in Xi'an city or nearby. There are detail inclusion/exclusion criteria: patients with cancer, infection, secondary IgAN (Secondary IgAN is seen most commonly in patients with liver disease or mucosal inflammation, in particular affecting the gastrointestinal tract), other renal diseases and autoimmune diseases were excluded. The exclusion criteria for healthy subjects included the chronic disease, central nervous system-related disease, and conditions involving vital organs (liver, heart, lung, brain) and more aggressive metabolic and endocrinological disease.

### SNP selection and genotyping

We examined 7 SNPs in *IL-1B* and 3 SNPs in *IL-6*, and all of the 10 SNPs had minor allele frequencies (MAF) greater than 5%. Samples were centrifuged and stored at −80° until analysis. We extracted genomic DNA from peripheral blood samples using the GoldMag-Mini Whole Blood Genomic DNA Purification Kit (GoldMag Ltd. Xi'an, China) according to the manufacturer's protocol and measured DNA concentrations using a NanoDrop 2000. Sequenom MassARRAY Assay Design 3.0 Software was used to design primers for amplification and extension reactions [33]. SNP genotyping was performed using a Sequenom MassARRAY RS1000 according to the manufacturer's standard protocol [33]. Finally, Sequenom Typer 4.0 Software was used for data management and analysis [33, 34].

### Statistical analysis

Departure from Hardy-Weinberg Equilibrium (HWE) was assessed for the frequency of each SNP in genotype frequencies between the esophageal cancer and control groups were evaluated using the Chi-square test [35]. Microsoft Excel and the SPSS 17.0 statistical package (SPSS, Chicago, IL) were used for statistical analyses. All P-values presented in this study are two-sided; P ≤ 0.05 was considered statistically significant. Odds ratios (OR) and 95% confidence intervals (CI) were calculated using unconditional logistic regression analyses [36]. The web-based software SNP Stats was used to identify associations between SNPs and the risk of esophageal cancer in four genetic models (Codominant, dominant, recessive, and additive) [37]. We used the Haploview software package (version 4.2) and the SHEsis software platform (http://analysis.bio-x.cn/myAnalysis.php) to analyze linkage disequilibrium, haplotype construction, and genetic associations at polymorphism loci [38, 39].

## References

[1] Berger J IgA glomerular deposits in renal disease. Transplant Proc.

[2] Berger J, Hinglais N (1968). Intercapillary deposits of IgA-IgG. [Article in French]. J Urol Nephrol (Paris).

[3] Alamartine E, Sabatier JC, Guerin C, Berliet JM, Berthoux F (1991). Prognostic factors in mesangial IgA glomerulonephritis: an extensive study with univariate and multivariate analyses. Am J Kidney Dis.

[4] Koyama A, Igarashi M, Kobayashi M, Research Group on Progressive Renal Diseases (1997). Natural history and risk factors for immunoglobulin A nephropathy in Japan. Am J Kidney Dis.

[5] Li PK, Ho KK, Szeto CC, Yu L, Lai FM (2002). Prognostic indicators of IgA nephropathy in the Chinese--clinical and pathological perspectives. Nephrol Dial Transplant.

[6] Donadio JV, Bergstralh EJ, Grande JP, Rademcher DM (2002). Proteinuria patterns and their association with subsequent end-stage renal disease in IgA nephropathy. Nephrol Dial Transplant.

[7] Syrjänen J, Mustonen J, Pasternack A (2000). Hypertriglyceridaemia and hyperuricaemia are risk factors for progression of IgA nephropathy. Nephrol Dial Transplant.

[8] Lim CS, Yoon HJ, Kim YS, Ahn C, Han JS, Kim S, Lee JS, Lee HS, Chae DW (2003). Clinicopathological correlation of intrarenal cytokines and chemokines in IgA nephropathy. Nephrology (Carlton).

[9] Harada K, Akai Y, Kurumatani N, Iwano M, Saito Y (2002). Prognostic value of urinary interleukin 6 in patients with IgA nephropathy: an 8-year follow-up study. Nephron.

[10] Eitner F, Westerhuis R, Burg M, Weinhold B, Gröne HJ, Ostendorf T, Rüther U, Koch KM, Rees AJ, Floege J (1997). Role of interleukin-6 in mediating mesangial cell proliferation and matrix production in vivo. Kidney Int.

[11] Novak J, Raskova Kafkova L, Suzuki H, Tomana M, Matousovic K, Brown R, Hall S, Sanders JT, Eison TM, Moldoveanu Z, Novak L, Novak Z, Mayne R (2011). IgA1 immune complexes from pediatric patients with IgA nephropathy activate cultured human mesangial cells. Nephrol Dial Transplant.

[12] Wu Y, Zhou BP (2009). Inflammation: a driving force speeds cancer metastasis. Cell Cycle.

[13] Dinarello CA (1996). Biologic basis for interleukin-1 in disease. Blood.

[14] Hsu SI, Ramirez SB, Winn MP, Bonventre JV, Owen WF (2000). Evidence for genetic factors in the development and progression of IgA nephropathy. Kidney Int.

[15] Galla JH (2001). Molecular genetics in IgA nephropathy. Nephron.

[16] Gharavi AG, Yan Y, Scolari F, Schena FP, Frasca GM, Ghiggeri GM, Cooper K, Amoroso A, Viola BF, Battini G, Caridi G, Canova C, Farhi A (2000). IgA nephropathy, the most common cause of glomerulonephritis, is linked to 6q22-23. Nat Genet.

[17] Gharavi AG, Kiryluk K, Choi M, Li Y, Hou P, Xie J, Sanna-Cherchi S, Men CJ, Julian BA, Wyatt RJ, Novak J, He JC, Wang H (2011). Genome-wide association study identifies susceptibility loci for IgA nephropathy. Nat Genet.

[18] Elinav E, Nowarski R, Thaiss CA, Hu B, Jin C, Flavell RA (2013). Inflammation-induced cancer: crosstalk between tumours, immune cells and microorganisms. Nat Rev Cancer.

[19] Ren K, Torres R (2009). Role of interleukin-1beta during pain and inflammation. Brain Res Brain Res Rev.

[20] Gemma C, Bickford PC (2007). Interleukin-1beta and caspase-1: players in the regulation of age-related cognitive dysfunction. Rev Neurosci.

[21] Friedman WJ (2001). Cytokines regulate expression of the type 1 interleukin-1 receptor in rat hippocampal neurons and glia. Exp Neurol.

[22] Paquette B, Therriault H, Wagner JR (2013). Role of interleukin-1β in radiation-enhancement of MDA-MB-231 breast cancer cell invasion. Radiat Res.

[23] Han J, Bae SY, Oh SJ, Lee J, Lee JH, Lee HC, Lee SK, Kil WH, Kim SW, Nam SJ (2014). Zerumbone suppresses IL-1β-induced cell migration and invasion by inhibiting IL-8 and MMP-3 expression in human triple-negative breast cancer cells. Chinese Journal of Rock Mechanics & Engineering.

[24] Chung ST, Geerts D, Roseman K, Renaud A, Connelly L (2017). Osteoprotegerin mediates tumor-promoting effects of Interleukin-1beta in breast cancer cells. Mol Cancer.

[25] Nakaya HI, Amaral PP, Louro R, Lopes A, Fachel AA, Moreira YB, El-Jundi TA, da Silva AM, Reis EM, Verjovski-Almeida S (2007). Genome mapping and expression analyses of human intronic noncoding RNAs reveal tissue-specific patterns and enrichment in genes related to regulation of transcription. Genome Biol.

[26] Leme Galvão LP, Menezes FE, Mendonca C, Barreto I, Alvim-Pereira C, Alvim-Pereira F, Gurgel R (2016). Analysis of association of clinical aspects and IL1B tagSNPs with severe preeclampsia. Hypertens Pregnancy.

[27] Sasayama D, Hori H, Teraishi T, Hattori K, Ota M, Iijima Y, Tatsumi M, Higuchi T, Amano N, Kunugi H (2011). Possible association between interleukin-1β gene and schizophrenia in a Japanese population. Behav Brain Funct.

[28] Sun GQ, Wu GD, Meng Y, Du B, Li YB (2014). IL-6 gene promoter polymorphisms and risk of coronary artery disease in a Chinese population. Genet Mol Res.

[29] Qi XF, Feng TJ, Yang P, Feng HY, Zhang P, Kong LY, Liang DL, Li PF, Na W, Li YW, Wang Y (2014). Role of inflammatory parameters in the susceptibility of cerebral thrombosis. Genet Mol Res.

[30] Du Y, Gao L, Zhang K, Wang J (2015). Association of the IL6 polymorphism rs1800796 with cancer risk: a meta-analysis. Genet Mol Res.

[31] Zheng X, Han C, Shan R, Zhang H, Zheng Z, Liu Y, Wang A (2015). Association of interleukin-6 polymorphisms with susceptibility to hepatocellular carcinoma. Int J Clin Exp Med.

[32] Donadio JV, Grande JP (2002). IgA nephropathy. N Engl J Med.

[33] Gabriel S, Ziaugra L, Tabbaa D (2009). SNP genotyping using the Sequenom MassARRAY iPLEX platform. Curr Protoc Hum Genet.

[34] Thomas RK, Baker AC, Debiasi RM, Winckler W, Laframboise T, Lin WM, Wang M, Feng W, Zander T, MacConaill L, Lee JC, Nicoletti R, Hatton C (2007). High-throughput oncogene mutation profiling in human cancer. Nat Genet.

[35] Adamec C (1964). EXAMPLE OF THE USE OF THE NONPARAMETRIC TEST. TEST X2 FOR COMPARISON OF 2 INDEPENDENT EXAMPLES. [Article in Czeck]. Cesk Zdrav.

[36] Bland JM, Altman DG (2000). Statistics notes. The odds ratio. BMJ.

[37] Solé X, Guinó E, Valls J, Iniesta R, Moreno V (2006). SNPStats: a web tool for the analysis of association studies. Bioinformatics.

[38] Barrett JC, Fry B, Maller J, Daly MJ (2005). Haploview: analysis and visualization of LD and haplotype maps. Bioinformatics.

[39] Shi YY, He L (2005). SHEsis, a powerful software platform for analyses of linkage disequilibrium, haplotype construction, and genetic association at polymorphism loci. Cell Res.

